# Influence of Farmer–Sheep Interactions in the Home Flock on Behaviour and Cortisol in a Communal Grazing Flock of Polish Mountain Sheep

**DOI:** 10.3390/ani16101447

**Published:** 2026-05-08

**Authors:** Paulina Nazar, Andrzej Junkuszew, Kamila Janicka, Monika Greguła-Kania

**Affiliations:** 1 Department of Animal Breeding and Agriculture Advisory, Faculty of Animal Sciences and Bioeconomy, University of Life Sciences in Lublin, 20-950 Lublin, Poland; paulina.nazar@up.edu.pl (P.N.); andrzej.junkuszew@up.edu.pl (A.J.); 2 Institute of Biological Basis of Animal Production, Faculty of Animal Sciences and Bioeconomy, University of Life Sciences in Lublin, 13 Akademicka Street, 20-950 Lublin, Poland; kamila.janicka@up.edu.pl

**Keywords:** behaviour, communal grazing system, human–animal interaction, positive animal welfare, sheep

## Abstract

Despite growing interest in human–animal interactions in livestock, limited information is available on whether differences in routine human contact in the home flock have lasting effects on sheep behaviour and physiological stress responses after transfer to a new herd. This study evaluated behavioural and cortisol related responses in 191 Polish Mountain sheep from five farms that were seasonally combined into one traditional Carpathian grazing flock. Before grazing, farms were classified according to selected characteristics of farmer contact with sheep, including, time spent with the flock, handling style, consistency of interaction, and farmer behaviour towards the animals. Sheep behavioural responses during milking were assessed by the shepherd and an independent observer using a five point scale in two observation periods and serum cortisol concentration was measured in ten sheep per farm. Behavioural scores were analysed using a cumulative link mixed model, with evaluator and season as fixed effects and farm and sheep identity nested within the farm as random effects. Descriptive mean behavioural scores ranged from 2.18 to 4.38, and mean cortisol concentrations ranged from 2.49 to 4.86. Farm level patterns suggested that sheep from farms with more favourable human contact tended to show calmer behaviour during milking and lower cortisol concentrations. These findings indicate that routine human contact in the home flock may be associated with later behavioural and physiological responses under communal grazing conditions.

## Simple Summary

Positive daily contact between farmers and sheep may shape how animals respond later in challenging management situations. This study examined whether experiences from the home flock affected the behaviour and stress of Polish Mountain sheep after animals from five farms were mixed into one communal grazing flock in the Carpathian pastoral system. Farms differed in the amount of time farmers spent with sheep and in the way animals were handled. Sheep behaviour during milking was assessed at the beginning and end of the grazing season, and blood cortisol was measured as an indicator of stress. Sheep from farms where contact with people was calmer and more positive behaved more quietly during milking and had lower cortisol concentrations than sheep from farms with poorer human contact. These differences were still observable at both observation periods during the grazing season, even though all animals were later kept under the same conditions. The findings suggest that previous experience with humans may be associated with later behavioural and physiological responses, rather than demonstrating a direct causal effect. Improving the way farmers handle and interact with sheep may contribute to reduced stress and facilitate handling, although other factors may also play a role.

## 1. Introduction

Animal husbandry is based on interspecies interdependence and often cooperation. Daily contact is inevitable and is shaped by human–animal interactions (HAI) [1]. As reported by Kling-Eveillard et al. [2], clearly defining these interactions is essential for efforts aimed at improving animal welfare in its broadest sense. In recent years, the concept of animal welfare has been extended beyond the prevention of negative states to include the promotion of positive experiences, such as comfort, play, exploration, and rewarding social contact [3,4,5,6]. The human–animal interaction is increasingly recognised as a central component of positive welfare, because regular, predictable, and gentle contact with humans can generate positive affective states and improve both welfare and productivity [4,6,7,8]. Understanding the mechanisms underlying the human–animal interactions is essential for developing management practices that minimise stress during human contact [9,10]. According to the model proposed by Hemsworth and Coleman [11], the attitudes of stockpeople towards animals influence handling behaviour, which in turn affects animals’ perception of humans and their behavioural and physiological responses related to stress and coping ability [12,13]. These responses to challenging situations may also impact animal productivity, highlighting the importance of identifying reliable welfare indicators [14]. Cortisol concentration in plasma or other tissues is widely used as an indicator of stress and animal welfare [15,16,17].

In the sheep sector, recent reviews have highlighted the need to consider positive aspects of welfare, including positive emotions and rewarding interactions with humans [18], and the important role of environmental factors and husbandry practices in shaping welfare, as shown in studies on goats [19] and sheep [20]. The human–sheep relationship is still less frequently considered in welfare assessments compared to other livestock species, although evidence suggests that positive interactions with humans, such as gentle handling, may reduce fear and agitation [21]. The behavioural responses of sheep towards humans depend to a significant extent on the environmental and social context [22]. Most studies on the human–animal interaction have been conducted in homogeneous flocks kept by a single farmer [1,23,24]. However, it remains unclear whether the effects of human–animal interactions persist when animals are transferred to a new environment and mixed with unfamiliar individuals, where both social structure and management conditions change, as regrouping and changes in group composition in sheep have been shown to alter behavioural responses, emotional state, and physiological parameters [25,26]. Previous studies have shown that animals’ responses to humans are shaped by prior experience and handling history; however, these responses may vary across contexts and are largely based on observations from stable production systems with consistent human contact [22].

Studies conducted in natural or semi-natural settings allow animals to express a broader behavioural repertoire, which may influence their responses to humans. Therefore, assessing HAI in conditions that reflect standard farming systems may provide more ecologically relevant information [27]. However, such approaches make it difficult to evaluate whether previous human experience continues to influence animal responses when individuals are exposed to new social and management conditions. The Carpathian communal grazing system may provide a valuable context for exploring whether previously established human–animal relationships remain reflected in behavioural and physiological responses under novel and socially dynamic conditions. This system is based on the seasonal grazing of several hundred sheep from different farmers in one large flock [28]. At the beginning of May, animals from different farmers are temporarily combined into a single flock and handed over to the care of a shepherd (baca) during the traditional mixing of the sheep. The animals graze on mountain pastures until October, after which they return to their home flocks for the winter. The sheep are kept in sheepfold buildings (indoor system) during the winter season [29,30,31].

The aim of this study was to evaluate whether previously experienced in the home flock human–animal interactions influence sheep behaviour and physiological responses after integration into a communal grazing flock. It was hypothesised that sheep originating from farms characterised by different types of human–animal interactions would differ in their behavioural responses during milking, as well as in cortisol levels, despite being kept under the same environmental conditions during the grazing period.

## 2. Materials and Methods

The study was conducted on a group of 191 sheep of a breed typical for the region, the Polish Mountain sheep. The observed animals grazed a mountain pasture in Beskid Śląski (49°33′45.7″ N, 18°54′59.7″ E) from May to October. The study was approved by the Local Ethics Committee for Animal Experimentation in Lublin, Poland (License No 104/2015).

### 2.1. Animal Management

In May, sheep from five different farmers (farms A–E) were combined into a single flock as part of traditional sheep mixing. The animals grazed the mountain pastures until the end of October, after which they were taken by the farmers to their home flocks [31]. The combined flock consisted of the shepherd’s sheep and those of four other farmers. In the present study, the term farmer refers to the owner of the home flock, locally known as gazda, whereas the communal flock during the grazing season was managed by a single shepherd. During the grazing period, the sheep were looked after by the shepherd with the help of two shepherd dogs.

### 2.2. Behavioural Observations

Before the start of the grazing season, farms were classified using a structured scoring system based exclusively on human-related factors describing farmer behaviour and interaction patterns with animals (Table 1). The classification included criteria reflecting both the quantity and quality of human–animal interactions, such as time spent with animals, handling style, consistency of interaction, and use of aversive or positive stimuli. Each criterion was scored on a three-point scale (1-low, 2-moderate, 3-high). The overall HAI score for each farm was calculated as the mean of all criteria, resulting in values ranging from 1 to 3. Based on this score, farms were classified into three categories: low HAI (1.00–1.66), moderate HAI (1.67–2.33), and high HAI (2.34–3.00). Higher HAI scores reflect more frequent, consistent, and positive human–animal interactions at the farm level (Table 2). Equal weighting of criteria was assumed due to the exploratory and field-based nature of the study. The scoring system was designed as a field-based proxy measure of HAI and was not formally validated. Similar multidimensional approaches to assessing the human–animal relationship at the farm level, based on stockperson-related variables and interaction patterns, have been described in the literature, although they differ in structure and level of validation [9,10]. These approaches typically integrate aspects such as handling behaviour, frequency of human contact, and consistency of interactions, which are considered key components of the human–animal relationship. Therefore, the applied scoring system should be interpreted as an approximation of handling conditions rather than a direct or validated measure of the human–animal interaction.

Observations of animals during grazing were conducted in two five-day cycles at the beginning of the grazing season (from 10 to 15 May) and at the end of the grazing season (from 2 to 7 October). In each cycle, observations were conducted for five consecutive days twice a day (in the morning at 8 a.m. and afternoon at 6 p.m.) during milking (performed by the shepherd). It took place in a designated area, where animals were gathered into a temporary enclosure. Sheep were handled individually and milked using a mobile milking unit. After milking, each animal immediately left the milking pen and returned to the pasture, maintaining constant visual and social contact with the flock. The vacated station was then taken by the next sheep. The animals entered the milking pen voluntarily. During the procedure, the observer recorded the behaviour of each sheep in terms of selected behavioural responses (Table 3). The behavioural scoring system was semi-quantitative in nature. The scoring focused on the intensity of resistance and the animal’s ability to remain in position during milking. Behavioural observations were conducted under extensive, pasture-based conditions within a communal grazing system, during routine milking on pasture, allowing the assessment of animal responses in a natural, non-experimental context without altering standard management practices, consistent with field-based welfare assessment approaches in dairy sheep [32]. The observer applied a predefined ethogram describing each behavioural category, and scoring was based on these predefined criteria rather than subjective real-time interpretation. Precise quantitative thresholds (e.g., frequency or duration of behaviours) were not defined.

**Figure 1:**
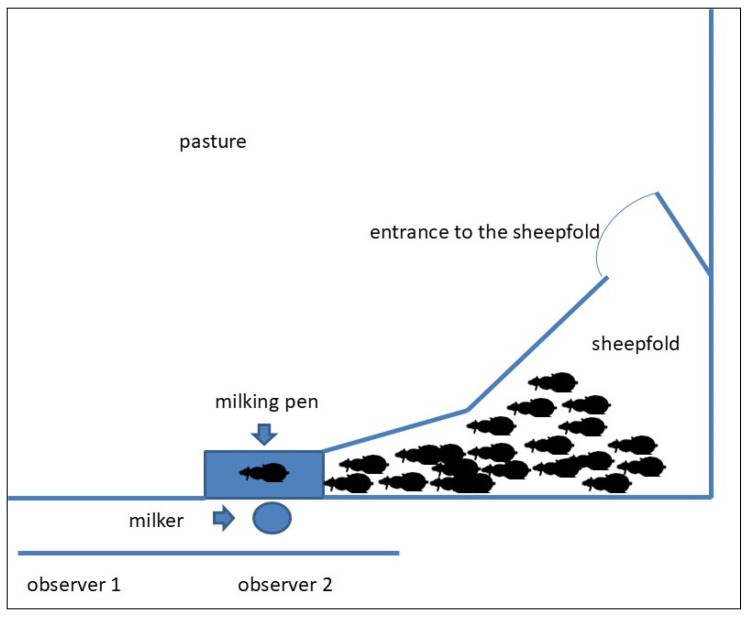
Scheme of the observation area during milking.

Behavioural observations were conducted during milking by two trained observers with a background in animal behaviour (ethology). One observer recorded the behaviour of each sheep, whilst the other noted the order in which the animals entered the milking pen. As only one observer performed behavioural scoring, inter-observer reliability was not assessed. The animals were identified using ear tags with individual numbers. Behaviour was assessed on a scale of 1 to 5 by both the shepherd and the observer. The observer evaluated behaviour based on predefined criteria (Table 3), whereas the shepherd independently provided a subjective assessment during milking using the same scoring scale. Based on the collected data, average behavioural scores were first calculated at the individual level and then averaged across the sheep from each farmer. To minimise potential observer bias, the observers were positioned in such a way as to allow undisturbed observation without direct contact with the milked animals (Figure 1).

### 2.3. Biochemical Analysis

Ten sheep were randomly selected from each farm. Immediately after milking, blood was collected from the jugular vein of each selected animal into tubes without anticoagulant to obtain serum. Blood samples were stored for 24 h at 4 °C and then centrifuged (2000× *g* for 30 min). The serum was stored at −20 °C and the cortisol level was determined.

The cortisol level was measured by an enhanced chemiluminescence enzyme using the IMMULITE 2000 (Siemens Healthcare Diagnostics Inc., Tarrytown, NY, USA) cortisol diagnostic test on an IMMULITE 2000 XPi SIEMENS analyser (Siemens Healthcare Diagnostics Inc., Tarrytown, NY, USA).

### 2.4. Statistical Analysis

Behavioural scores were ordinal variables on a 1 to 5 scale and were therefore analysed using a cumulative link mixed model with a logit link function (CLMM). Evaluator and season were included as fixed effects, while farm and sheep identity nested within the farm were included as random effects to account for the clustering of animals within farms and the repeated observations of the same sheep. The model structure was:

Behavioural score = evaluator + season + (1|farm) + (1|farm:sheep_id)

The significance of fixed effects was assessed using likelihood ratio tests. Descriptive behavioural data are presented as mean ± SE according to farm and evaluator, whereas statistical inference was based on the mixed ordinal model.

Cortisol concentrations are presented as mean ± SD by farm. Associations between behavioural scores, order of entry, cortisol concentration, and farm assessment were analysed using Spearman rank correlations calculated from farm level aggregated data to avoid pseudoreplication. Therefore, one farm represented one statistical unit in correlation analyses. Due to the small number of farms, these correlations were interpreted as exploratory.

All analyses were performed in R (version 4.6.0).

## 3. Results

The cumulative link mixed model showed the significant effect of the evaluator on behavioural scores during milking (LR = 23.34, df = 1, *p* < 0.001), whereas the effect of the season was not significant (LR = 0.77, df = 1, *p* = 0.380) (Table 4). This indicates that behavioural scores differed between the observer and the shepherd, while no significant differences were found between the two observation periods.

The random effects included farm and sheep identity nested within the farm, thereby accounting for the clustered structure of the data and repeated behavioural assessments of the same animals (Table 5).

Descriptive behavioural scores according to farm of origin are presented in Table 6. The highest mean behavioural scores, both for the observer and the shepherd, were observed in sheep from farms A and B (A-4.27 and 4.36, B-4.38 and 4.37), whereas the lowest scores were recorded in sheep from farm E (1.87 and 2.50). These farm-based differences are presented descriptively, because statistical inference for behavioural scores was based on the cumulative link mixed model.

Cortisol concentrations according to farm of origin are presented in Table 7. The lowest mean cortisol concentration was observed in sheep from farm A (2.49), while the highest mean value was recorded in sheep from farm E (4.86). Intermediate concentrations were found in sheep from farms B (3.41), C (3.77) and D (4.66).

**Figure 2:**
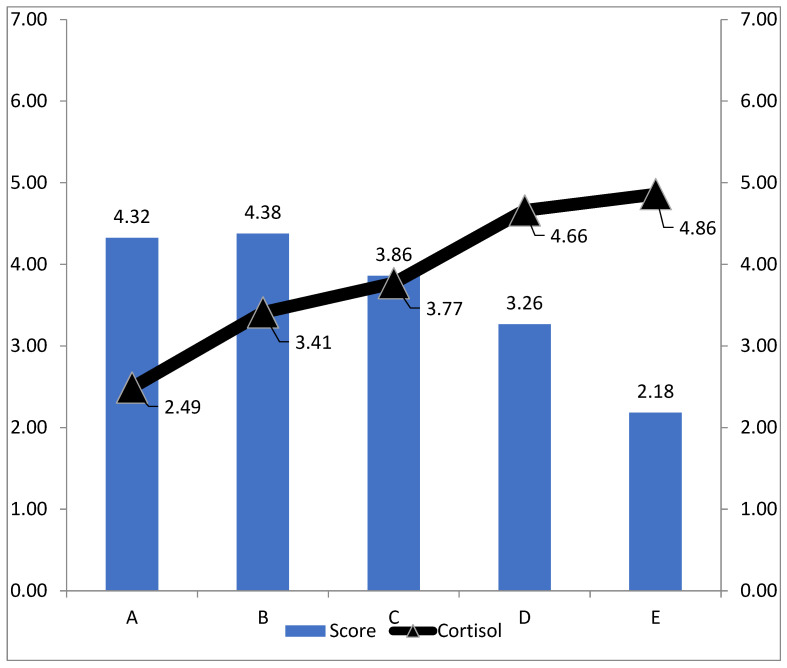
Mean ± SE behavioural score and mean ± SE cortisol concentration by farm of origin. Behavioural score was calculated as the mean of observer and shepherd scores for graphical presentation only. The figure is descriptive; statistical inference for behavioural scores was based on the cumulative link mixed model.

For graphical presentation, behavioural values were averaged across observer and shepherd assessments and are presented in Figure 2 together with mean cortisol levels. Sheep from farm A showed the lowest cortisol levels (2.49), whereas the highest values were observed in sheep from farm E (4.86), with intermediate values in farms B (3.41), C (3.77), and D (4.66). Overall, higher behavioural scores were associated with lower cortisol levels. Sheep from farms A and B, which had the highest behavioural scores (4.32 and 4.38, respectively), also showed the lowest cortisol levels, whereas sheep from farm E, with the lowest behavioural score (2.18), exhibited the highest cortisol concentration (4.86).

Farm level Spearman correlations are presented in Table 8. Strong positive associations were observed between shepherd and observer behavioural scores, while negative associations were observed between behavioural scores and both order of entry and cortisol concentration. The strongest association was found between shepherd assessed behavioural score and mean cortisol concentration (ρ = −1.00, N = 5, *p* = 0.017). However, because correlations were calculated using farm level aggregated data and the number of farms was limited, these associations should be interpreted as exploratory.

## 4. Discussion

Dairy production systems involve frequent human–animal contact, making it a relevant context for studying the effects of human–animal interactions. Animals with more positive human experiences show greater willingness to interact and are easier to handle [2,10,33]. Importantly, high-quality human–animal relationships not only reduce fear but also promote positive affective states, which may translate into improved welfare and productivity [4,6]. In the present study, conducted under the Carpathian communal grazing system, where animals from different farms were combined into a single flock, descriptive farm level patterns suggested that sheep from farms with more favourable human–animal interaction scores tended to show calmer behaviour during milking and lower cortisol concentrations within the communal flock. Similar relationships between human contact, behavioural responses, and physiological stress indicators have been reported in sheep and other livestock species [8,9,33,34]. However, previous studies also indicate that these relationships are not always consistent and may depend on environmental and management conditions, suggesting that the effects of human–animal interactions may vary across production systems. Nevertheless, these findings should be interpreted with caution, as the observed behavioural and physiological patterns may be influenced by multiple interacting factors. In addition, regrouping and changes in group composition are known to affect behavioural and emotional responses in sheep, suggesting that such responses are context-dependent [25,26]. It is also possible that differences between farms reflect not only variation in human–animal interactions, but also other farm-level factors, such as management practices or early-life conditions.

The farm level association between shepherd and observer assessments suggested that both evaluators captured a similar general pattern of behavioural variation among farms. However, a significant evaluator effect was observed, suggesting systematic differences in scoring between the observer and the shepherd. Therefore, the two assessment sources should be interpreted as complementary rather than fully interchangeable. That observation is consistent with previous studies showing that stockperson-based assessments can reflect animals’ responses to handling [1,9]. Descriptive differences between farms may be associated with variation in behavioural responses in the new communal flock. However, because the analysis accounted for differences between farms, these patterns should be interpreted as farm-level tendencies rather than direct post hoc comparisons between farms. In contrast, no significant seasonal effect was detected, indicating that behavioural scores did not change substantially between the two observation periods under the conditions of this study. Behavioural responses in sheep may vary depending on context and are not always temporally stable, as shown in previous studies [22]. This suggests that the persistence of differences observed in the present study may not be universal and could be influenced by specific characteristics of the studied system, including its extensive and socially dynamic nature. This may support the notion that earlier human-related experiences may be associated with persistent behavioural differences observed across the grazing season [6,34]. Consequently, one of the factors that may contribute to the observed differences between farms is the quality of human–animal interactions in the home flock, rather than the shared environmental conditions in the communal grazing system. At the same time, this interpretation should be treated with caution, as the relative contribution of human–animal interactions compared to other farm-level factors cannot be fully disentangled in the present study. This may be related to the fact that the animals stayed on the home farms from late autumn to early spring and were kept in an indoor system. During this period, the farmer has daily contact with the sheep for feeding and routine management procedures such as shearing, hoof trimming, deworming, as well as during lambing and the rearing of lambs. The amount of time devoted to the animals and the quality of interactions with the farmer may therefore influence their responses to humans. As reported by Kruger et al. [19], when contact with the animals is limited to feeding and technical procedures only, animal welfare may be impaired. Therefore, it is important to build positive relationships during daily animal handling, where the farmer can calmly touch and speak to the animals, as gentle tactile contact has been shown to modulate physiological responses and reduce stress in sheep [7]. The effects of such interactions may, however, vary depending on their consistency and the broader management context. In contrast, negative interactions cause fear and distrust [2,9,12].

Recent reviews of sheep welfare highlight that housing conditions, nutrition, health management, and social environment interact closely with stockperson behaviour and handling practices [8,20]. At the same time, these studies emphasise that isolating the specific contribution of human–animal interactions remains challenging due to the complexity of production systems. This aligns with earlier findings indicating that human–animal interactions are an important determinant of animals’ behavioural and physiological responses [9,12,34]. Although extensive mountain grazing systems are generally considered beneficial due to access to pasture and the opportunity to express natural behaviours, they may also expose animals to environmental challenges [20]. The present results suggest that, even under shared grazing conditions, farm level differences were reflected in descriptive behavioural patterns and in cortisol concentrations. However, these patterns should be interpreted with caution as associations rather than direct causal effects, as the contribution of human–animal interactions cannot be fully separated from other farm-level factors and may also reflect pre-existing variation between farms. Therefore, the observed relationships should be interpreted as associations rather than direct causal effects. This suggests that stockperson-related factors may represent an important component of welfare outcomes, alongside environmental conditions [6]. Within the framework of positive animal welfare, these findings may indicate that regular, calm and predictable contact with humans may contribute not only to reduced fear but also to more stable behavioural responses [3,4]. At the same time, the extent to which these effects are maintained under varying environmental and management conditions remains uncertain.

Studies in the sheep sector indicate that farmers often associate good welfare primarily with health and grazing conditions, while the role of human–animal interactions in shaping affective states is less frequently recognised [18]. In this context, the classification of farms applied in the present study may represent a practical tool linking scientific concepts of welfare with on-farm practice. Emphasising the benefits of calm and predictable handling may support both welfare improvement and easier animal management, as animals that are less fearful of humans are known to be easier to handle and more cooperative during routine procedures [9]. The observed relationships between behavioural scores and the order of entry into the milking pen may suggest that previous human–animal interactions are associated with social dynamics within the flock. In the present study, differences in human–animal interaction at the farm level may be associated with variation in behavioural responses of sheep. These differences may also translate into interactions between animals. Behavioural traits related to human reactivity may also reflect broader aspects of animal temperament, which can influence social interactions within the group, as suggested in previous studies on sheep [22]. Given that social relationships were not assessed directly in the present study, this interpretation requires cautious interpretation. According to Guhl and Atkeson [35], access to resources can reflect hierarchical position. In line with this, sheep from farms with lower behavioural scores consistently entered the milking pen later, which may indicate a lower social position. As the order of entry represents only an indirect measure, this interpretation should be made with caution. This may suggest that human–animal interactions could be associated not only with responses to humans but also with aspects of social organisation within the flock.

From a broader perspective, traditional communal grazing systems in the Carpathian region are recognised for their cultural and ecological importance [28,29,30,36]. While such systems provide favourable conditions for the expression of natural behaviours, the present results indicate that environmental factors alone may be insufficient to ensure optimal welfare. The persistence of behavioural and physiological differences between animals from different farms throughout the grazing season may suggest that early human-related experiences are associated with sustained differences in behavioural and physiological responses under these conditions [6,34]. At the same time, these observations may also reflect interactions between environmental conditions and management practices within the system, rather than the isolated effect of human–animal interactions. Integrating traditional grazing systems with improved human–animal interactions may therefore be an important direction for enhancing welfare in extensive production systems. These findings have practical implications for livestock management. Improving the quality of human–animal interactions at the level of home farms may contribute to reduced stress and more stable behaviour in animals, which may in turn facilitate handling and reduce labour requirements. However, changing stockperson behaviour remains challenging, as it is influenced by long-established habits and attitudes [34,37]. Therefore, strategies aimed at improving animal welfare should combine scientific knowledge with practical communication, highlighting the tangible benefits of improved handling for farmers, including easier management and reduced animal stress.

Several limitations of the present study should be considered. A comprehensive assessment of human–animal interactions ideally requires consideration of both human- and animal-related components of the interaction [9,33]. In the present study, the HAI classification focused exclusively on human-related factors, reflecting farmer behaviour during routine handling and daily management practices. While this approach provides a practical representation of handling conditions at the farm level, it does not capture the full bidirectional nature of the human–animal relationship and should therefore be interpreted as a simplified proxy measure rather than a validated assessment tool. In addition, potential confounding factors, such as differences in management practices, nutrition, genetics, and early-life conditions between farms, cannot be fully excluded and may have contributed to the observed differences [8]. These factors should be considered when interpreting the results, particularly given the observational nature of the study. Furthermore, the hierarchical structure of the data, with animals nested within farms, should be taken into account. Although the analyses accounted for the hierarchical structure of the data, the limited number of farms restricted the strength of farm-level inference, particularly for associations involving farm assessment. Behavioural assessment partly relied on the shepherd’s subjective evaluation during milking. While less standardised, this reflects practical perception of animal behaviour under farm conditions and was consistent with the observer-based scoring. In addition, the communal flock was managed by a single shepherd, who was also the owner of one of the flocks; however, this ensured the consistency of handling conditions throughout the grazing season. Another limitation concerns the assessment of cortisol. Cortisol concentrations were determined in a relatively small number of animals per farm and at a single sampling time point following milking. Since cortisol is a dynamic marker that may be affected by acute handling-related stress and temporal physiological variation, these measurements should be interpreted as a snapshot of the animals’ physiological state rather than a comprehensive indicator of baseline stress [17]. Finally, social relationships were not assessed directly, and the order of entry into the milking pen represents only an indirect measure, which limits the interpretation of social dynamics.

## 5. Conclusions

In summary, the results of the present study indicate that human–animal interactions in the home flock may play an important role in shaping sheep behaviour and physiological responses after integration into a communal grazing system. Sheep originating from farms characterised by more favourable human–animal interaction scores tended to show calmer behaviour during milking and lower cortisol levels. This suggests that previous experience with humans may have a lasting influence on animals’ responses in a new social and environmental context. However, these findings should be interpreted as associations rather than direct causal effects, and may also be influenced by farm-level factors and other contextual variables. From a practical perspective, improving the quality of human–animal interactions at the level of home flocks may contribute to more stable behaviour and facilitate handling under extensive production conditions. Further research is needed to better understand the relative contribution of human–animal interactions and other farm-level factors, as well as their long-term influence across different production systems.

## Figures and Tables

**Table 1:** Criteria used to approximate human–animal interaction (HAI) at the farm level.

Criterion	Score 1 (Low)	Score 2 (Moderate)	Score 3 (High)	Rationale
Time spent with animals	<1 h/day	1–2 h/day	>2 h/day	Reflects frequency of human exposure, which influences habituation and familiarity with humans
Presence beyond routine tasks	Only for feeding/technical tasks	Limited additional presence	Regular calm presence	Indicates non-threatening human exposure, promoting habituation
Handling style	Forceful, rough (e.g., shouting, pushing)	Neutral, routine handling	Calm, gentle handling	Represents quality of human behaviour, a key determinant of fear vs. trust
Handling during procedures	Forceful restraint	Moderate control	Calm, low-stress handling	Reflects human behaviour during stressful events, directly relevant to outcomes like milking behaviour
Consistency of interaction	Unpredictable handling	Moderately consistent	Highly consistent routine	Indicates predictability, which affects stress and learning in animals
Use of aversive stimuli	Frequent use (e.g., hitting, loud noise)	Occasional use	Minimal or none	Reflects negative human impact, associated with fear and stress responses
Positive tactile contact	Rare or none	Occasional	Frequent, calm touching	Represents positive reinforcement, linked to reduced stress and improved human–animal interactions

**Table 2:** Classification of farms based on human–animal interaction (HAI) scores.

Farm	Mean HAI Score	HAI Category
A *	3.00	high
B	2.71	high
C	1.84	moderate
D	2.00	moderate
E	1.14	low

*—shepherd was the owner of flock A; HAI—human–animal interaction.

**Table 3:** Detailed criteria for assessing sheep behaviour during milking.

Score	Behavioural Description	Operational Definition	Ease of Milking
5	Calm and fully cooperative	No resistance; stands still; no kicking	No difficulty
4	Mild reactivity	Occasional minor movements (e.g., slight leg lifting); remains cooperative	Uninterrupted
3	Moderate restlessness	Intermittent kicking, head movements; manageable handling	Slightly affected; completed without interruption
2	High reactivity	Frequent resistance (kicking, backing away); difficulty maintain position	Difficult
1	Strong aversive response	Refusal to enter; escape attempts, intense struggling; lying down	Difficult or interrupted

**Table 4:** Likelihood ratio tests for fixed effects in the cumulative link mixed model of behavioural scores.

Effect	LR Statistic	df	*p*-Value
evaluator	23.34	1	<0.001
season	0.77	1	0.380

Behavioural scores were analysed using a cumulative link mixed model. Evaluator and season were included as fixed effects, while farm and sheep identity nested within the farm were included as random effects. LR, likelihood ratio statistic; df, degrees of freedom.

**Table 5:** Random effects structure of the cumulative link mixed model of behavioural scores.

Random_Effect	SD	Variance
Sheep identity nested within farm Farm	4.25	18.09
	3.11	9.69

SD—standard deviation. Farm and sheep identity nested within the farm were included as random effects to account for the clustering of sheep within farms and the repeated behavioural assessments of the same animals.

**Table 6:** Descriptive behavioural scores of sheep during milking according to farm of origin (mean ± SE).

Farm	*N*	Observer	Shepherd
A	162	4.28 ± 0.07	4.37 ± 0.05
B	60	4.38 ± 0.10	4.37 ± 0.10
C	74	3.68 ± 0.15	4.04 ± 0.10
D	34	3.00 ± 0.14	3.53 ± 1.18
E	52	1.87 ± 0.29	2.50 ± 1.26

Data are presented as mean ± SE. N denotes the number of pooled observations from the beginning and end of the grazing season.

**Table 7:** Cortisol concentration in sheep according to farm of origin (mean ± SD).

Farm	*N*	Cortisol
A	10	2.49 ± 0.95
B	10	3.41 ± 1.22
C	10	3.77 ± 1.40
D	10	4.66 ± 1.19
E	10	4.86 ± 1.09

N-number of blood samples.

**Table 8:** Spearman rank correlations among farm level mean behavioural scores, mean order of entry, mean cortisol concentration and farm assessment.

Variable_1	Variable_2	Spearman’s ρ	N	*p*-Values
shepherd	observer	0.9	5	0.083
shepherd	order entry	−0.9	5	0.083
shepherd	farm assessment	0.9	5	0.083
shepherd	cortisol mean	−1.0	5	0.017
observer	order entry	−0.8	5	0.133
observer	farm assessment	0.8	5	0.133
observer	cortisol mean	−0.9	5	0.083
order entry	farm assessment	−0.7	5	0.233
order entry	cortisol	0.9	5	0.083
farm assessment	cortisol	−0.9	5	0.083

N corresponds to the number of farms.

## Data Availability

The data presented in this study are available on request from the corresponding author.
